# The role of sclerostin and dickkopf-1 in oral tissues – A review from the perspective of the dental disciplines

**DOI:** 10.12688/f1000research.17801.1

**Published:** 2019-01-30

**Authors:** Mohammad Samiei, Klara Janjić, Barbara Cvikl, Andreas Moritz, Hermann Agis

**Affiliations:** 1 Department of Endodontics, Faculty of Dentistry, Tabriz University of Medical Sciences, Tabriz, Iran; 2 Department of Conservative Dentistry and Periodontology, University Clinic of Dentistry, Medical University of Vienna, Vienna, 1090, Austria; 3 Austrian Cluster for Tissue Regeneration, Vienna, 1200, Austria

**Keywords:** Sclerostin, Dickkopf-1, Wnt Pathway, Regeneration, Periodontology, Endodontology

## Abstract

Wnt signaling is of high relevance in the development, homeostasis, and regeneration of oral tissues. Therefore, Wnt signaling is considered to be a potential target for therapeutic strategies. The action of Wnt is tightly controlled by the inhibitors sclerostin (SOST) and Dickkopf (DKK)-1. Given the impact of SOST and DKK-1 in hard tissue formation, related diseases and healing, it is of high relevance to understand their role in oral tissues. The clinical relevance of this knowledge is further underlined by systemic and local approaches which are currently in development for treating a variety of diseases such as osteoporosis and inflammatory hard tissue resorption. In this narrative review, we summarize the current knowledge and understanding on the Wnt signaling inhibitors SOST and DKK-1, and their role in physiology, pathology, and regeneration in oral tissues. We present this role from the perspective of the different specialties in dentistry, including endodontics, orthodontics, periodontics, and oral surgery.

## Introduction

Research and development of novel treatment strategies for regenerative dentistry has become crucial to improve oral health due to the growing numbers of dental and maxillofacial problems which require treatment. Therefore, cell-therapy modalities, biologicals, and gene therapy approaches are in development with the aim to provide novel tools for the clinical demands ( Dissanayaka *et al*., 2014; Fretwurst *et al*., 2018; Itoh *et al*., 2018; Kaigler *et al*., 2015; Nevins *et al*., 2005; Plonka *et al*., 2017; Taut *et al*., 2013). This research needs to be guided by an appropriate understanding of the cell biological mechanisms underlying pathological changes and regeneration in the oral tissue, including the periodontium and the dental tissue. Due to the major role of Wnt signaling and the respective regulators in development and healing, research in regenerative medicine and dentistry has evaluated the feasibility of targeting the pathway for therapeutic approaches ( Florio *et al*., 2016; Heiland *et al*., 2010; Taut *et al*., 2013; Yu *et al*., 2018).

**Figure 1.**
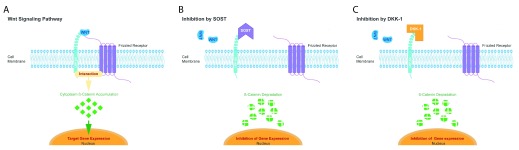
Inhibition of Wnt signaling by sclerostin and dickkopf-1. Scheme of the Wnt signaling pathway ( **A**) which is inhibited by sclerostin (SOST, **B**) and dickkopf-1 (DKK-1, **C**). Adopted from ( Yorgan & Schinke, 2014) and modified.

The Wnts comprise a family of at least 19 lipid-modified glycoproteins and short-range ligands. Wnts can activate canonical and non-canonical pathways of signaling in the cells ( Mohammed *et al*., 2016; Tang *et al*., 2009; Yang *et al*., 2016). The canonical Wnt path involves the interaction of Wnt with frizzled (Frz) and low density lipoprotein receptor-related protein 5/6 (LRP5/6). Thereby β-catenin accumulation is induced leading to the translocation of β-catenin into the nucleus where target genes are activated ( Mohammed *et al*., 2016; Tang *et al*., 2009; Yang *et al*., 2016) ( Figure 1).

Wnt signaling regulates various cellular functions which include cell migration, proliferation, differentiation, apoptosis, and morphogenesis. Given this broad involvement of Wnt signaling it is not surprising that it has a key role in organ development, regeneration and homeostasis of tissues ( Logan & Nusse, 2004; Sarkar & Sharpe, 2000; Seo *et al*., 2012). In the maxillofacial region, Wnt signaling modulates morphological patterns in tooth development including teeth number, shape, size, and positioning ( Han *et al*., 2011; Kim *et al*., 2013; Kratochwil *et al*., 2002; Liu *et al*., 2008; Sarkar *et al*., 2000; Wang *et al*., 2009; Zhang *et al*., 2013). Maturation of dental mesenchyme into odontoblasts and cementoblasts is also modulated by Wnt signaling which highlights the relevance to dentistry ( Janjić *et al*., 2018; Zhang *et al*., 2013). Given this dominant role of Wnts, Wnt signaling needs to be tightly controlled involving inhibitors.

Sclerostin (SOST) and dickkopf-1 (DKK-1) are the primary inhibitors controlling the Wnt signaling pathways ( Figure 1). These inhibitors can directly bind to LRP5/6 and inhibit the activation of LRP5- and LRP6- related signaling. Dkk-1 binds to a larger region on LRP5 and LRP6 extracellular surface and thereby can inhibit binding with other Wnts than SOST. ( Ahn *et al*., 2011; Bourhis *et al*., 2010; Bourhis *et al*., 2011; Cheng *et al*., 2011; Florio *et al*., 2016; Li *et al*., 2005; van Bezooijen *et al*., 2004) Thereby SOST and DKK-1 play critical roles in the formation of hard tissue and associated diseases. SOST and DKK-1 are consequently considered potential therapeutic targets for regenerative approaches ( Janjić *et al*., 2018; Li *et al*., 2005; Semënov *et al*., 2005; Taut *et al*., 2013).

The glycoprotein SOST is mainly secreted by osteocytes, the mechanosensor cells of the bone, and has a major effect on bone and dental tissue ( Kuchler *et al*., 2014; Pflanz *et al*., 2017; Saag *et al*., 2017; Taut *et al*., 2013; Winkler *et al*., 2003; Yu *et al*., 2018). In recent years, SOST production in oral tissues has gained more attention and led to *in vitro* and *in vivo* studies on the regulation of SOST ( Pflanz *et al*., 2017; Taut *et al*., 2013; Yu *et al*., 2018). SOST production in mineralizing periodontal ligament cells is increased; this observation suggests that targeting of SOST might support periodontal regeneration. SOST has also been demonstrated to have a role in the dimension of the periodontal ligament ( Kuchler *et al*., 2014). This further supports the hypothesis of a key role of SOST in oral tissues. DKK-1, another antagonist of Wnt signaling, modulates the dimension of oral tissues, including the periodontium and teeth ( Bao *et al*., 2013; Jäger *et al*., 2010; MacDonald *et al*., 2007).

**Figure 2.**
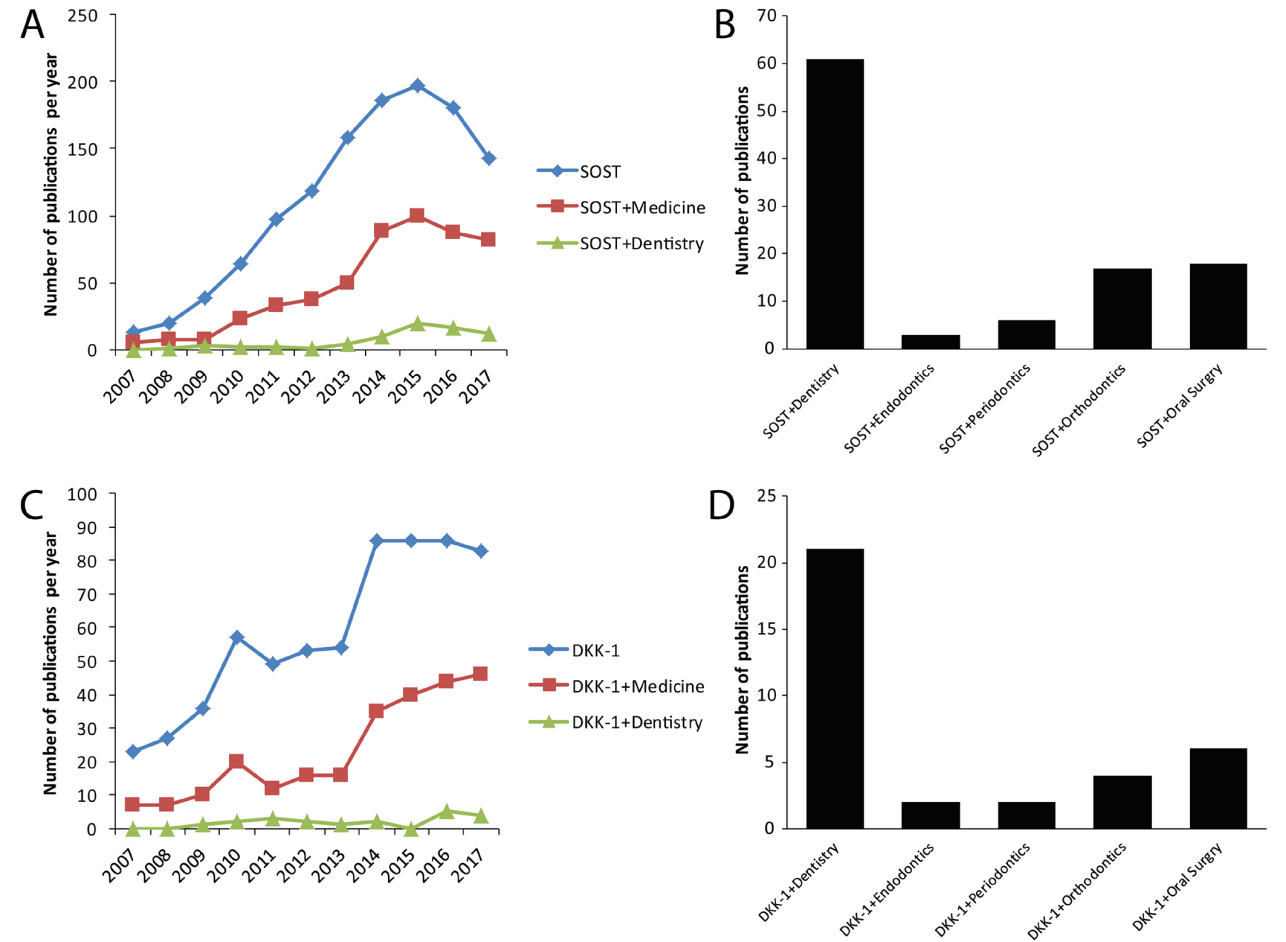
The numbers of publication on sclerostin and dickkopf-1 increased rapidly over the past 10 years. The number of publications on sclerostin (SOST, **A**) and dickkopf-1 (DKK-1, **C**) per year as found in Pubmed.org (Search terms SOST or DKK-1 with and without Medicine or Dentistry). The total number of publications on SOST ( **B**) and DKK-1 ( **D**) in Dentistry and the respective specialties Endodontics, Periodontics, Orthodontics, and Oral Surgery as found in Pubmed.org (Search terms SOST with and without Dentistry, Endodontics, Periodontics, Orthodontics, or Oral Surgery).

According to the findings of 10 years analysis of publications regarding SOST and DKK-1 in medical fields ( Figure 2), it becomes evident that there is a growing set of literature on SOST and DKK-1. Within dentistry the majority of publications on SOST and DKK-1 is from the field of oral surgery. This review aims to provide a synopsis of the existing knowledge on the Wnt signaling inhibitors SOST and DKK-1 from endodontics, orthodontics, periodontics, and oral surgery perspectives.

## Endodontics perspective

Research and developments in endodontics lead to the continuous improvement of the field, as the introduction of new techniques and biomaterials drive profound progress in endodontic clinical practice. In particular regenerative endodontics is an exciting dental specialty. Treatment protocols of infected root canals in immature teeth were established which allow continued apical closure and root maturation ( Chueh & Huang, 2006; Huang, 2008; Huang *et al*., 2008). In these regenerative endodontic procedures, after treatment with ethylenediaminetetraacetic acid (EDTA) signaling factors embedded in the dentin matrix such as TGF-β are exposed and released into the canal space ( Galler *et al*., 2015). These factors can guide the migration of dental pulp stem cells and support differentiation into odontoblast-like cells, leading to the production of mineralized matrix also termed reparative dentin ( Nakashima *et al*., 1994; Rutherford *et al*., 1993; Sloan & Smith, 1999; Tziafas *et al*., 1998). Interestingly TGF-β has the capacity to induce SOST expression ( Gruber *et al*., 2017; Manokawinchoke *et al*., 2015); therefore, it is possible that dentin conditioning can interfere with the regulation system of Wnt signaling ( Gruber *et al*., 2017; Manokawinchoke *et al*., 2015).

Clinically applied regenerative protocols, such as revascularization, utilize the regenerative capacity of endogenous stem cells. During treatment, over instrumentation into the periapical tissues causes stem cells to migrate and enter into the canal system via the blood clot. This process leads to the treatment of immature teeth with pulp necrosis by replacing dentin, root structures and pulp-dentin complex cells ( Diogenes *et al*., 2016). However, revascularization can be accompanied by intracanal calcification ( Song *et al*., 2017). It is unclear how Wnts and their inhibitors SOST and DKK-1 are involved in this process. Therefore, studies which investigate the regulation of SOST and DKK-1 in pulp cells and tissue under conditions which are present in the early phase of healing are of clear relevance ( Janjić *et al*., 2018).

Results of Zhang *et al*. indicate that Wnt/β-catenin signaling is required for odontoblastic differentiation and also promotes proliferation of pre-odontoblasts and odontogenesis during root development ( Zhang *et al*., 2013). Based on these findings, the integrity of Wnt/β-catenin signaling in odontoblasts is vital for proliferation and differentiation through root formation. Targeted deletion of β-catenin in odontoblasts leads to incomplete incisors and rootless molars. Furthermore, β-catenin deficiency disrupts the differentiation of odontoblasts and cementoblasts. ( Zhang *et al*., 2013) Epithelial expression of DKK-1 or epithelium-specific inactivation of β-catenin causes abnormal tooth development at the early bud stage ( Andl *et al*., 2002; Chen *et al*., 2009; Han *et al*., 2011). Mesenchyme-specific inhibition of β-catenin indicates the critical role of Wnt/β-catenin signaling in the potential mesenchymal odontogenic activation throughout early tooth growth ( Chen *et al*., 2009).

After tooth development odontoblasts secrete dentin and the pulp chamber system narrows with age ( Arana-Chavez & Massa, 2004; Foster *et al*., 2013; Lee *et al*., 2013; Sakai *et al*., 2010). While dentin gradually thickens, the pulp chamber space is reduced and a massive bone loss can be observed during aging. ( Boskey & Coleman, 2010; Carvalho & Lussi, 2017; Gabet & Bab, 2011) In response to injuries and stress the formation of mineralized tissue by odontoblasts, also termed reparative dentinogenesis, is stimulated. The odontoblasts which are involved in this repair process are clearly responsive to Wnt ( Zhao *et al*., 2018). SOST knockout mice demonstrate dramatically enhanced formation of reparative mineralized bridges and increased mineralization in dental pulp cells compared with wild-type mice ( Collignon *et al*., 2017). These findings are related to an increased SOST expression in wild type cells. Further more these results show that SOST deficiency accelerates reparative dentinogenesis after pulp damage and therefore inhibition of SOST may provide a promising therapeutic strategy to improve the healing of injured pulps ( Collignon *et al*., 2017).

Expression of DKK-1 is up-regulated in a rodent model of induced periapical lesions, suggesting that DKK-1 is involved in the inflammatory processes and bone resorption in periapical lesions ( Zhang *et al*., 2014). Interestingly several potential therapeutic approaches rely on the modulation of Wnt signaling ( Ishimoto *et al*., 2015; Lee *et al*., 2016; Zhao *et al*., 2018), including several plant-derived molecules. Baicalein promotes odontoblastic differentiation and angiogenesis of human dental pulp cells. It was suggested that via the inhibition of DKK-1 Baicalein can contribute to regenerative endodontics and dental pulp repair. ( Lee *et al*., 2016).

Given the importance of hypoxia-induced signaling in the early phase of pulp healing we investigated the production of SOST and DKK-1 in dental pulp cells upon treatment with hypoxia or the hypoxia mimetic agent L-mimosine in monolayer, spheroid, and tooth slice cultures. Our results show that the response with regard to SOST and DKK-1 production depends on the culture model.

Taken together, the literature highlights a major role of SOST and DKK-1 in tooth development and as a potential target for regenerative strategies.

## Orthodontics perspective

Orthodontic tooth movement is a result of external force applications to the teeth, an intervention which involves remodeling in dental and surrounding oral tissues, such as periodontal ligament, alveolar bone, and gingiva ( Antoun *et al*., 2017; Krishnan & Davidovitch, 2006). The applied forces induce displacement of the teeth in the periodontal ligament space, thereby establishing sites where the tissue is compressed and sites were traction establishes ( Antoun *et al*., 2017; Krishnan & Davidovitch, 2006). Thereby, modeling of the alveolar bone through the processes of bone resorption and bone formation is induced leading to clinical changes in the position of the tooth. ( Krishnan & Davidovitch, 2006; Krishnan & Davidovitch, 2009; Reitan, 1967)

Mechanical forces can regulate the expression of SOST by osteocytes; for instance the expression of SOST is reduced when loading is increased, and increased when loading is decreased leading to an increase in bone formation or bone loss, respectively ( Moustafa *et al*., 2012; Robling *et al*., 2008). This feature of SOST indicates that it is a crucial protein in bone formation under mechanical stimulation ( Agholme *et al*., 2011; Mantila Roosa *et al*., 2011; Robling *et al*., 2008; ). Given that SOST is produced by osteocytes and cementocytes in alveolar bone and in cellular cementum, respectively, an involvement of SOST in tooth movement is likely ( Jäger *et al*., 2010).

During orthodontic tooth movement, one of the essential processes is remodeling of the periodontal ligament. Periostin is one of the factors which increased in the periodontal ligament during initial stages of orthodontic tooth movement ( Wilde *et al*., 2003), a matricellular protein and collagen-rich tissues which are highly expressed in periosteum under persistent mechanical stress ( Horiuchi *et al*., 1999). Periodontal cells have shown to respond to these mechanical forces with an increase of TGF-β which can stimulate SOST ( Gruber *et al*., 2017; Manokawinchoke *et al*., 2015). The increase in the levels of SOST in the compression side and the decrease in the tension side during tooth movement can be suggestive of an interplay between periostin from Sharpey’s fibres and SOST in alveolar bone during orthodontic therapy ( Nishiyama *et al*., 2015).

Cementoblasts are highly differentiated cells from the mesenchymal lineage which generate cementum ( Bosshardt, 2005). Diseases that influence bone characteristics often interfere with the properties of the cementum, which highlights the similarities of these tissues. Although SOST is expressed in both osteocytes and cementocytes in dental tissues ( Jäger *et al*., 2010), it is still not clear if cementocytes can have similar mechanical stress sensing capacity as osteocytes. However, SOST which is released by osteocytes in the alveolar bone may affect the function of cementoblasts. This process might be modulated upon orthodontic treatment. Research on the role of SOST can also help to understand the impact of therapeutics on the periodontium. Strontium supports differentiation of cementoblasts ( Bao *et al*., 2014) with one possible underlying mechanism being that strontium decreases the production of SOST. Thus, strontium was proposed for regenerative approaches to support cementum production in cases of root resorption. ( Bao *et al*., 2014) However, if SOST or DKK-1 can serve effectively as targets for orthodontic approaches and cementum repair requires further studies.

## Periodontics perspective

Periodontitis is an inflammatory disease of the teeth supporting tissues, has a multifactorial etiology. The inflammation created by specific microorganisms extends deep into the tissues and causes the destruction of the tooth supporting connective tissue and alveolar bone. This progressive process leads to the pathological impairment of collagen fibres, loss of periodontal ligament and alveolar bone recession ( Armitage, 2004; Highfield, 2009; Pihlstrom *et al*., 2005). Evaluation of SOST and DKK-1 in chronic periodontitis patients showed that both of these inhibitors were up-regulated in the periodontal tissues of these subjects ( Napimoga *et al*., 2014).

There are hints from animal studies that inflammation can not only trigger bone resorption, but also decrease bone formation. This anti-anabolic effects seem to be mediated by increased levels of DKK-1 ( Heiland *et al*., 2010). *In vitro* models of oral soft tissue augmentation suggest that DKK-1 is also increased in oral soft tissue wound healing ( Agis *et al*., 2014). Thus, Wnt inhibitors can play a major role in pathological processes and regeneration in the periodontal tissue. Although periodontitis can be treated in early stages, mostly because of the chronic entity of problem, it is diagnosed in the advanced phase of destruction of the periodontal ligament and the prognosis of maintaining the teeth are poor. Conventional regenerative approaches use biomaterials of natural or synthetic origin as filler for the defect thereby aiding the host to replace lost periodontal tissue and bone. While these interventions can stimulate tissue repair and stop the destruction of the periodontium, methods to archive full regeneration are still the focus of research ( Hernández-Monjaraz *et al*., 2018).

The blocking of Wnt signaling impairs the periodontal ligament and alveolar bone ( Lim *et al*., 2014), while enhancing Wnt signalling by SOST knock out stimulates alveolar bone formation and reduces the width of periodontal ligament ( Kuchler *et al*., 2014). Expression of SOST by cementocytes suggests that these cells may regulate cell activity on the cementum surface ( Bao *et al*., 2013; Lehnen *et al*., 2012). TGF-β can increase the production of SOST in fibroblasts from periodontal ligament and gingiva ( Gruber *et al*., 2017). This mechanism seems to be involved in the impact of mechanical loading on mineralized tissue formation in the periodontal ligament ( Manokawinchoke *et al*., 2015). Deletion of SOST leads to more cellular cementum, in parallel to more dramatically increased alveolar bone deposition ( Kuchler *et al*., 2014). Blocking SOST by application of a SOST-specific antibody enhances healing of alveolar bone in experimental periodontitis ( Chen *et al*., 2015; Liu *et al*., 2018; Taut *et al*., 2013). In addition, it was reported that reduced SOST in periostin knockout mice can re-establish periodontal ligament and alveolar bone ( Rangiani *et al*., 2016; Ren *et al*., 2015). This evidence supports that targeting of SOST is a feasible approach for periodontal therapy.

Dental cementum is a mineralized hard tissue on the surface of root dentin and present either in acellular or cellular form. Defective cementum results in periodontal breakdown, tooth dysfunction, and finally leads to tooth loss. Cementogenesis is a key element in the process of periodontal tissue regeneration ( Bosshardt, 2005; Kao & Fiorellini, 2012). SOST was detected only in cementocytes of cellular cementum in the late stages of cementum development ( Lehnen *et al*., 2012). SOST levels in cementocytes increased in periodontal ligament cultures, following mineralization treatment ( Jäger *et al*., 2010). Interestingly, in periodontal ligament cells Baicalein can promote osteoblastic differentiation involving Wnt/β-catenin signaling ( Chen *et al*., 2017). DKK-1 significantly reversed the effects of Baicalein on human periodontal ligament cells ( Chen *et al*., 2017). It is possible that this mechanism can be exploited in regenerative approaches.

The here presented literature supports the significant effects of SOST and DKK-1 in the periodontium system and periodontal diseases. As a result, they could be the main targets in future periodontics regenerative therapies.

## Oral surgery perspective

The alveolar bone supports the tooth in the maxilla and mandible and is characterized by continuous and rapid remodeling in response to mechanical forces ( Javed *et al*., 2010; Pagni *et al*., 2012). Thereby alveolar bone continuously adapts to functional load. If this mechanical stimuli is lacking the alveolar bone undergoes a resorptive process ( Einhorn & Gerstenfeld, 2015; Pagni *et al*., 2012; Sodek & McKee, 2000). Following trauma due to overloading or surgery bone has the capacity to regenerate. While long bone healing occurs by endo-chondral ossification, alveolar bone healing typically occurs without histological cartilage formation ( Devlin *et al*., 1997). The success of oral surgery procedures, such as implants, depends on the proper healing of alveolar bone and strategies which stimulate bone regeneration ( Lin *et al*., 2011). Thus understanding the cell and molecular biological background of bone healing is clearly of clinical relevance.

In bone, SOST is mainly secreted by osteocytes and represents a key modulator of bone homeostasis ( Brunkow *et al*., 2001; van Bezooijen *et al*., 2004). The importance of SOST in bone formation is illustrated by sclerosteosis, a rare autosomal recessive disorder with a loss-of-function mutation in SOST ( Sebastian & Loots, 2018; Yavropoulou *et al*., 2014). Further evidence comes from Van Buchem Disease, which is characterized by a noncoding deletion which removes a SOST-specific regulator ( Sebastian & Loots, 2018; Yavropoulou *et al*., 2014). These diseases show bone overgrowth, particularly in the craniofacial bones and the jaw bone ( Balemans *et al*., 2002; Brunkow *et al*., 2001). There is also a phenotype in oral tissue; partial anodontia, malocclusion, and delayed tooth eruption is seen in subjects with Van Buchem Disease or SOST ( Stephen *et al*., 2001; van Bezooijen *et al*., 2009). Animal studies on the role of SOST in loss of function or gain function models indicate SOST decreases bone formation and can stimulate bone resorption ( Canalis, 2013; Li *et al*., 2008; O’Brien *et al*., 2013). SOST knockout mice show a dramatically increased basal mandibular bone and less effect on the coronal and apical part of the alveolar bone ( Kuchler *et al*., 2014).

The response of bone to mechanical loading is highly important in implantology. Since SOST is a key player in the regulation of bone formation in the response to mechanical loading it is important to understand its role in the alveolar bone. Upon loading, osteocytes reduce the expression of SOST permitting Wnts to bind their receptors. ( Burgers & Williams, 2013; Galli *et al*., 2010; Zhao *et al*., 2013). Interestingly, SOST expression is up-regulated around implants without primary stability ( Shu *et al*., 2017). Thus, it is very likely that SOST regulates the adaptation of bone around dental implants to the mechanical forces of loading. Application of SOST specific antibodies has been shown to stimulate bone formation around dental implants ( Yu *et al*., 2018).

DKK-1 can decrease Wnt/β catenin signalling and reduced the production of type II collagen in chondrocytes under mechanical loading ( Niu *et al*., 2016). Interestingly, DKK-1 has been found to play a distinct role in inflammation-induced bone loss ( Heiland *et al*., 2010). However, when evaluating genetic markers for peri-implantitis clinically, no direct relation of DKK-1 and peri-implantitis is seen ( Hall *et al*., 2011). Thus, further research is required to understand the role of DKK-1 in the alveolar bone.

From a clinical standpoint, osteoporosis is a risk factor in implantology. Inspired by the fact that inhibition of SOST can effectively improve bone formation in osteoporotic patients strategies which harness this capacity to improve bone formation around implants have been evaluated. ( Canalis, 2013; Costa & Bilezikian, 2012; Recker *et al*., 2015; Shu *et al*., 2017). Implant osseointegration is superior in the SOST knockout mice suggesting that SOST is a promising target to enhance implant osseointegration in osteoporosis. ( Shu *et al*., 2017) Also application of antibodies specific for SOST can improve bone formation around implants ( Yu *et al*., 2018).

Taken together these data show the relevance of the Wnt signalling inhibitors of SOST and DKK-1 for implantology and bone augmentation, as well as for tumor development and cancer therapy. Future studies will show how effective targeting of SOST and DKK-1 can be applied to stimulate regeneration and for tumor therapy.

## Conclusion

The here presented review highlights the relevance of Wnt signaling, the inhibitors SOST and DKK-1, and its key role in oral tissue homeostasis throughout life ( Florio *et al*., 2016; Heiland *et al*., 2010; Witcher *et al*., 2018). Major sources of Wnt inhibitors are the mechanosensors of the bone, the osteocytes ( Moustafa *et al*., 2012; Odagaki *et al*., 2018; Robling *et al*., 2008). Cells from oral tissue including cementocytes can modulate Wnt signaling by these inhibitors and mice deficient of SOST or DKK-1 have highlighted the important role in the oral tissue ( Jäger *et al*., 2010; Lehnen *et al*., 2012). Based on this knowledge concepts to antagonize inhibitors Wnt signaling have been developed to support oral tissue regeneration ( Taut *et al*., 2013; Yu *et al*., 2018). The future will reveal the capacity of these strategies which target these Wnt signaling inhibitors for regenerative therapy in dentistry. Given the interplay of SOST and DKK-1, a bidirectional approach which targets both SOST and DKK-1 locally has high potential ( Florio *et al*., 2016).

## Data availability

No data is associated with this article.
