# Supplementary material for: The role of sclerostin and dickkopf-1 in oral tissues – A review from the perspective of the dental disciplines
Source: F1000Res. 2019 Jan 30;8:128. [Version 1] doi: 10.12688/f1000research.17801.1 (PMC6468704; doi:10.12688/f1000research.17801.1)
Supplement: Peer review history

# Peer review: Reviewer response for version 1

**DOI:** 10.5256/f1000research.19462.r46090

**Published:** 2019-04-15

**Authors:** Qiming Jin

**Affiliations:** 1 Department of Cariology, Restorative Sciences, and Endodontics, School of Dentistry, University of Michigan, Ann Arbor, MI, USA

This manuscript is a review paper on sclerostin and dickkopf-1 impacts on dentistry. The authors made good publication search work, and reviewed the expressions and biological functions of sclerostin and DKK-1 in each parts of tooth and surrounding tissues. Furthermore, the authors explored the correlation between SOST and DKK-1 and various pathologies. More importantly, the treatment pathways to target SOST and DKK-1 were discussed profoundly. The only flaw is the lack of SOST and DKK-1 in enamel formation, which does not affect the merits of this paper.

I have read this submission. I believe that I have an appropriate level of expertise to confirm that it is of an acceptable scientific standard.

# Peer review: Reviewer response for version 1

**DOI:** 10.5256/f1000research.19462.r43827

**Published:** 2019-02-04

**Authors:** Francisco Javier Rodríguez-Lozano

**Affiliations:** 1 Cellular Therapy and Hematopoietic Transplant Unit, Hematology Department, Virgen de la Arrixaca Clinical University Hospital, IMIB-Arrixaca, University of Murcia, Murcia, Spain

The manuscript fits into the aim of the journal. The manuscript is well written and the results presented interesting.

 I would like to know the search strategy. Inclusion/exclusion criteria.

 Minor aspects:

 Endodontic perspective: "Given the importance of hypoxia-induced signaling in the early phase of pulp healing we investigated the production of SOST and DKK-1 in dental pulp cells upon treatment with hypoxia or the hypoxia mimetic agent L-mimosine in monolayer, spheroid, and tooth slice cultures". Citation?

 Periodontic Perspective: " Dental cementum is a mineralized hard tissue on the surface of root dentin and present either in acellular or cellular form. Defective cementum results in periodontal breakdown, tooth dysfunction, and finally leads to tooth loss." I recommend adding a reference.

 Finally, I recommend re-writing the conclusion without citations

I have read this submission. I believe that I have an appropriate level of expertise to confirm that it is of an acceptable scientific standard.
